# A Standardized Diagnostic Pathway for Suspected Appendicitis in Children Reduces Unnecessary Imaging

**DOI:** 10.1097/pq9.0000000000000541

**Published:** 2022-03-30

**Authors:** Roshan J. D’Cruz, Allison F. Linden, Courtney L. Devin, Jillian Savage, Arezoo Zomorrodi, Kirk W. Reichard, Arabinda Choudhary, Loren Berman

**Affiliations:** From the 1 Department of Pediatric General Surgery, Nemours Children’s Health, Wilmington, Del.; 2 Sidney Kimmel Medical College at Thomas Jefferson University Hospital, Philadelphia, Pa.; 3 Department of Emergency Medicine, Nemours Children’s Health, Wilmington, Del.; 4 Department of Radiology, University of Arkansas for Medical Sciences, Little Rock, Ark.

## Abstract

Ultrasound (US) for the diagnosis of acute appendicitis is often nondiagnostic, and additional imaging is required. A standardized approach may reduce unnecessary imaging.

**Methods:**

We retrospectively analyzed all patients who had imaging for appendicitis in our emergency department in 2017 and evaluated patient characteristics associated with nondiagnostic US. Using these results, we developed a pediatric appendicitis score (PAS)-based imaging pathway and compared imaging trends prepathway and postpathway implementation.

**Results:**

A total of 971 patients received imaging for suspected appendicitis prepathway in 2017. Female sex, obesity, and low/intermediate PAS were significantly associated with nondiagnostic US, but not magnetic resonance imaging (MRI) (*P* < 0.0001). Nearly one-third of patients received multiple imaging studies (US followed by MRI/computed tomography). As low/intermediate PAS was most strongly associated with a nondiagnostic US on multivariate analysis, we developed a PAS-based imaging stewardship pathway to eliminate imaging in low-PAS patients and reduce the number of patients with an intermediate PAS who received multiple imaging studies by obtaining an MRI as the first-line study. After implementation, only 22 low-PAS patients received imaging (compared with 238 preimplementation), and the proportion of intermediate-PAS patients receiving multiple imaging studies decreased from 31.4% to 13% (*P* < 0.0001). The cost of imaging per 100 patients increased from $24,255 to $31,082.

**Conclusion:**

A PAS-based imaging stewardship pathway reduces unnecessary imaging for suspected appendicitis.

## INTRODUCTION

Appendectomy is the most common acute pediatric general surgical procedure performed in the United States.^1^ The approach to the diagnosis of appendicitis has evolved with the increased availability of imaging. Clinical scoring systems that incorporate clinical symptoms, physical examination signs, and laboratory results were originally developed to aid in diagnosis^2,3^; however, today they are primarily used to determine which patients benefit from imaging.^4^

The American College of Radiology recommends ultrasound (US) as the first-line imaging modality in children with suspected appendicitis.^5^ The accuracy of US is excellent when the appendix is definitely visualized,^6^ but the appendix is often not visualized,^7^ leading to additional imaging. There has been a move away from computed tomography (CT) in children because of the ionizing radiation and concern for associated malignancies.^8^ Magnetic resonance imaging (MRI) has become a popular confirmatory study and has comparable accuracy with CT without exposure to ionizing radiation.^9–11^ Despite current recommendations, the ideal imaging strategy is debatable, particularly for individuals at high risk for a nondiagnostic US, or in institutions with high rates of nondiagnostic US. Attempts at using clinical risk assessment tools similar to the pediatric appendicitis score (PAS) to guide imaging decisions have occurred but no consensus has been reached.^12,13^ The ease of availability and the perception that US is a relatively low-cost study have resulted in high reliance on US to “rule out” appendicitis.

We sought to identify patient characteristics associated with nondiagnostic US at our hospital. We then used the PAS to develop a pathway to guide the workup of patients with suspected appendicitis to create a consistent approach to decision-making intended to reduce unnecessary imaging for children with suspected appendicitis.

## METHODS

### Determination of Factors Associated with a Nondiagnostic Imaging Study

We performed a retrospective chart review of patients who underwent imaging for suspected appendicitis at our hospital between January 1, 2017, and December 31, 2017. The team obtained demographic data and imaging results from the electronic medical record. The PAS was retrospectively calculated through chart review and categorized into low (1–3), intermediate (4–6), or high (7–10) risk for appendicitis.^14^ The ultimate diagnosis was determined by chart review.

### Definitions

A diagnostic US study required a fully visualized appendix and a report of normal (negative) or inflamed (positive). A nondiagnostic US did not visualize, or only partly visualized, the appendix, irrespective of secondary signs. We defined a positive MRI as a visualized enlarged appendix with secondary signs of inflammation in the right lower quadrant (RLQ). A negative MRI was either a visualized noninflamed appendix with no secondary signs of inflammation in the RLQ or a nonvisualized appendix with no secondary signs of inflammation in the RLQ. We considered an MRI nondiagnostic if the appendix was not fully visualized, but there were secondary signs of appendicitis, such as free fluid in the RLQ, or a mildly enlarged noninflamed appendix with no secondary signs of inflammation. A negative appendectomy was defined based on pathological findings as the absence of histological evidence of transmural inflammation. The team defined a missed appendicitis as a patient who was discharged from the emergency department (ED) with no suspicion for appendicitis and returned with appendicitis within 48 hours.

### Statistical Analysis

Associations between patient characteristics and having nondiagnostic US or MRI were tested using chi-square or Fisher exact test, as appropriate. We used multivariate analysis to identify which patient characteristics were associated with nondiagnostic imaging after adjusting for covariates. All variables with *P* value <0.10 on bivariate analysis, in addition to demographic characteristics, were included in the model. The team used these findings to develop a PAS-based imaging pathway.

### Creation and Implementation of a PAS-based Imaging Pathway

**Fig. 1.**
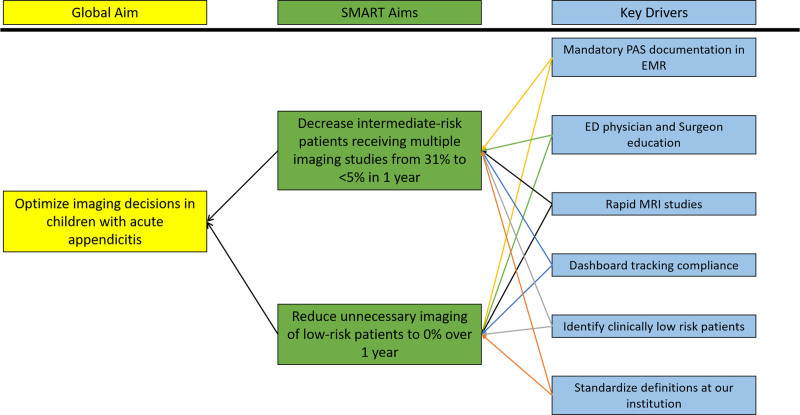
Key driver diagram outlining our objective, SMART aims, and the related key drivers. EMR, electronic medical record; WBC, white blood cells.

**Fig. 2.**
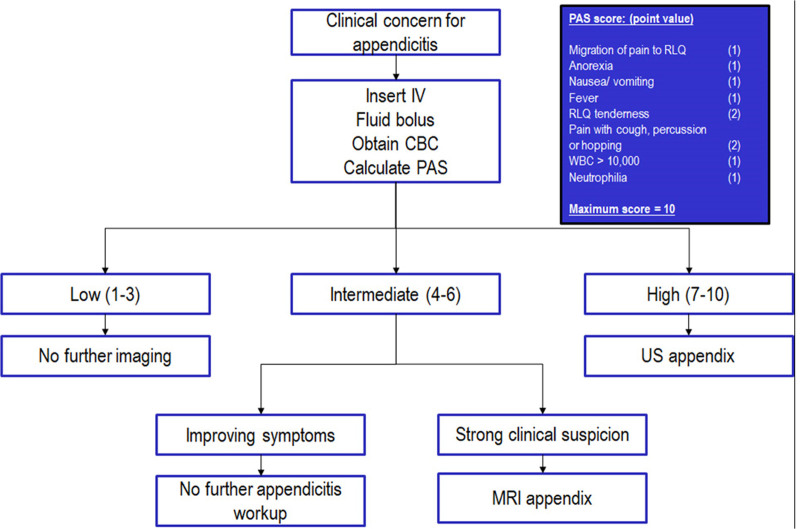
Imaging pathway. Flow diagram created to determine imaging study based on PAS. CBC, complete blood count; IV, Intravenous line.

We convened a multidisciplinary team including surgery, emergency medicine, and radiology to address overutilization of imaging studies in children with suspected appendicitis. The stakeholder group identified key drivers (Fig. 1), and developed a standardized imaging protocol for children in the ED being evaluated for suspected appendicitis. The pathway (Fig. 2) went into effect on April 1, 2019. A CT was obtained if the patient did not tolerate MRI or if perforated appendicitis was suspected on the initial US in the high-risk group.

We incorporated a PAS calculator into the electronic medical record and an ED-specific appendicitis order set, including laboratory tests, surgery consultation, pain control, and recommended imaging. Documentation of PAS by the ED physician was required and embedded within the appendicitis imaging order. The ED physician was also prompted to reassess intermediate-PAS patients with a repeat physical examination, recalculate the PAS following a fluid bolus, and review of laboratory studies’ results. The team implemented the pathway following education to all emergency room staff and surgeons. PAS does not appear in the radiologist’s view of the imaging requisition, so it was unlikely to affect study interpretation.

All patients presenting to the ED for the 9-month period (April–December 2019) after implementing the pathway and having a PAS score documented were analyzed. Patients transferred to our ED after imaging at an outside hospital were excluded.

### Statistical Analysis

#### Comparison of Historical Controls (2017) with Postimplementation Data

We used the historical control group from 2017 to compare imaging patterns according to PAS group pre and postimplementation. We used the 2017 patients because their PAS scores were retrospectively calculated, and we did not have PAS data on 2018–2019 patients preimplementation. We quantified the proportion of patients requiring multiple imaging studies (US and MRI, US and CT, or all three studies) according to the PAS group (low, intermediate, and high) and compared retrospective data from 2017 with postimplementation data from 2019. The chi-square test was used to compare the proportion of patients who received multiple imaging studies in the two different periods. For patients with low PAS, we compared the absolute number of imaging studies performed in this group after implementation of the imaging pathway with the same 9-month period in 2017. We could not compare proportions since there was no way to calculate the denominator for the patients in 2017, as they were selected based solely on the fact that they had undergone imaging to rule out appendicitis. The negative appendectomy rate and incidence of missed appendicitis were also compared using chi-square testing.

#### Comparison of Immediate Pre and Postimplementation Data

Using statistical process control methodology, we tracked the incidence of multiple imaging studies in all patients independent of PAS status in the time frame immediately pre and postimplementation. We also tracked room to disposition time to evaluate whether changes in imaging patterns impacted ED throughput. Room to disposition decision time controls for variability in ED throughput related to volume by adjusting for waiting time for an available room on the inpatient ward. Control limits were set at three sigma from the mean. Standard rules were applied to determine if changes were due to common or special cause variation. MRI rate (defined as proportion of all patients undergoing evaluation for suspected appendicitis who received an MRI) was calculated pre and postimplementation. We measured imaging costs per 100 patients immediately pre and postimplementation to assess the pathway’s impact on cost. Statistical process control charts were created using QI-charts V.2.0.23 software (Scoville Associates, Cambridge, MA, 2009) for Microsoft Excel (Microsoft, Redmond, WA, 2016). All other statistics were performed using SAS Enterprise Guide v 5.1 (Cary, NC).

The project was deemed a quality improvement project by our Institutional Review Board. We used SQUIRE methodology in the design and description of this study.^15^

## RESULTS

### Performance of US and MRI in 2017 and Clinical Outcomes by PAS

In 2017, there were 971 unique ED patient encounters with imaging performed for suspected appendicitis, including 939 US, 250 MRI, and 78 CT scans. The overall nondiagnostic rate was 72.9% for US and 9.2% for MRI. Female sex, obesity, and a low/intermediate PAS were significantly associated with a nondiagnostic US (*P* < 0.0001), but no factors were associated with a nondiagnostic MRI (Table 1). On multivariable analysis adjusting for age, sex, race, payor status, and obesity, a low-PAS patient was 3.4 times more likely than a high-PAS patient to have a nondiagnostic US (odds ratio 3.4, 95% confidence interval 2.1–5.3, *P* < 0.0001). Patients with intermediate PAS were 2.3 times more likely than high-PAS patients to have a nondiagnostic US (odds ratio 2.3, 95% confidence interval 1.6–3.4, *P* < 0.0001).

**Table 1.** Associations between Patient Characteristics and Nondiagnostic US and MRI Study (2017)

	% Nondiagnostic US (No. Patients)	*P*	% Nondiagnostic MRI (No. Patients)	*P*
Age range		0.12		0.69
Younger than 5 y	81.5% (75)		0	
5–10 y	70.7% (220)		9.6% (7)	
Older than 10 y	72.9% (392)		9.4% (16)	
Sex		<0.0001		0.59
Male	66.4% (286)		8.1% (9)	
Female	78.6% (401)		10.1% (14)	
Race		0.13		0.34
White	71% (444)		7.4% (12)	
African American	79.8% (103)		11.1% (4)	
Asian/Indian	65% (13)		0%	
Other	76% (127)		15.2% (7)	
Ethnicity		0.43		0.28
Hispanic	76.4% (139)		13% (7)	
Non-Hispanic	72.2% (547)		8.2% (16)	
Payor		0.07		0.20
Private	71.9% (425)		7.3% (11)	
Public	76.3% (245)		13.2% (12)	
Self-pay	58.6% (17)		0	
Weight-for-age category		<0.0001		0.72
Normal	68.2% (371)		10.6% (14)	
Overweight	75.2% (124)		7.9% (3)	
Obese	82.8% (192)		7.5% (6)	
PAS		<0.0001		0.60
1–3 (low)	81% (209)		5.7% (3)	
4–6 (intermediate)	74.2% (389)		10.2% (15)	
7–10 (high)	55.8% (88)		10% (5)	

**Table 1, Supplemental Digital Content 1,** http://links.lww.com/PQ9/A363, summarizes clinical outcomes stratified by PAS. The low-PAS group accounted for 268 patient encounters that had imaging for appendicitis. Of these, 79.8% (218) of patients were discharged from the ED without further workup, and 2% (7) of patients were admitted for observation. The incidence of appendicitis in the low-PAS group was 0.7%, whereas the intermediate- and high-PAS groups had higher rates of appendicitis (15% and 60%, respectively).

### Pathway Implementation and Imaging Studies Performance

**Fig. 3.**
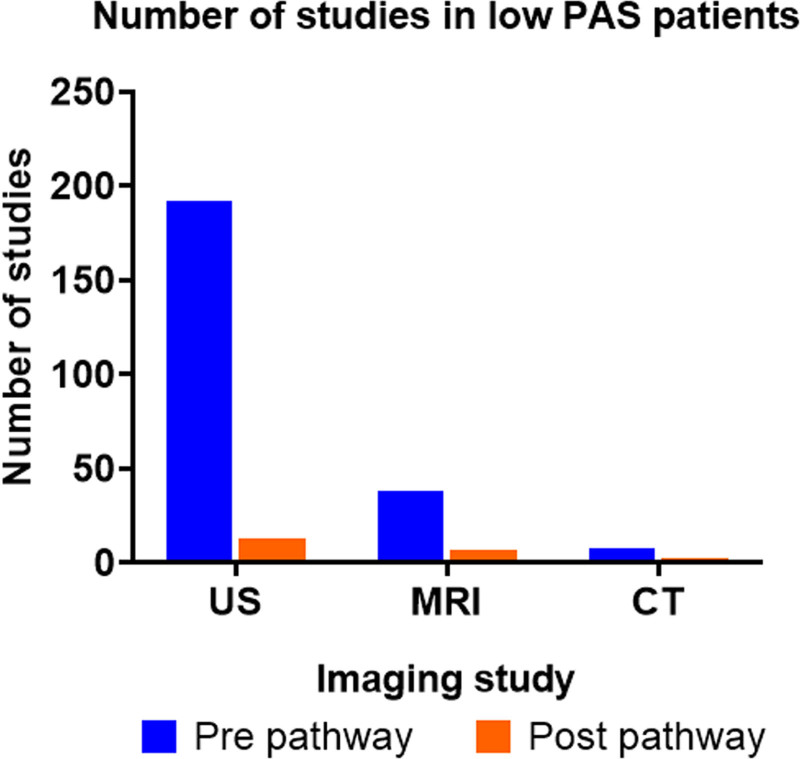
Comparison of pre and postimplementation imaging utilization. Number of imaging studies (US, MRI, and CT scans) performed pre and postimplementation of the pathway in the low-PAS group.

A total of 437 patients were evaluated for appendicitis in our ED who had a PAS documented in the 9 months postpathway implementation (April–December 2019) (**see Figure 1, Supplemental Digital Content 3,** which describes patient breakdown into PAS groups for pre and postimplementation cohorts, http://links.lww.com/PQ9/A365). There were no significant differences between the pre and postimplementation groups regarding age, weight, sex, or race (**see Table 2, Supplemental Digital Content 2,** http://links.lww.com/PQ9/A364). For patients with a low-PAS score, Figure 3 summarizes the number of imaging studies performed in the same 9-month periods (April–December) in 2017 and 2019. Briefly, from April to December 2017, 238 imaging studies were performed (192 US, 38 MRI, and 8 CT scans) and 73.8% of the US were nondiagnostic. After pathway implementation (April–December 2019), 22 studies were performed in the low-PAS group (13 US, 7 MRI, and 2 CT scans).

**Fig. 4.**
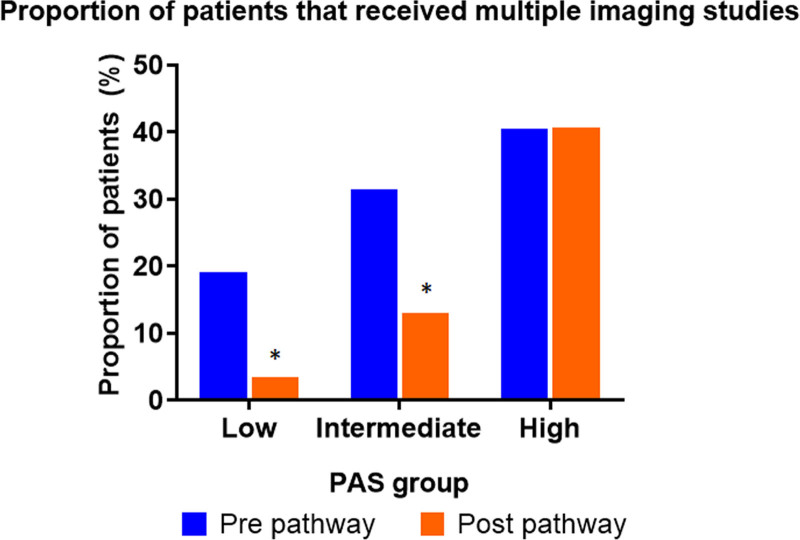
Proportion of patients who had multiple imaging studies pre and postimplementation of the pathway for intermediate-and high-PAS groups (*P < 0.0001).

Implementation of the pathway resulted in a reduction in the proportion of patients receiving multiple imaging studies in the low- and intermediate-PAS groups (low PAS, 19%–3.4% [*P* < 0.0001]; intermediate PAS, 31.4%–13.0% [*P* < 0.0001]). However, in the high-PAS group, where US was the initial study of choice, the proportion of patients who received multiple imaging studies remained high and not significantly different at 40.6% versus 40.4% (*P* = 0.97) (Fig. 4).

**Fig. 5.**
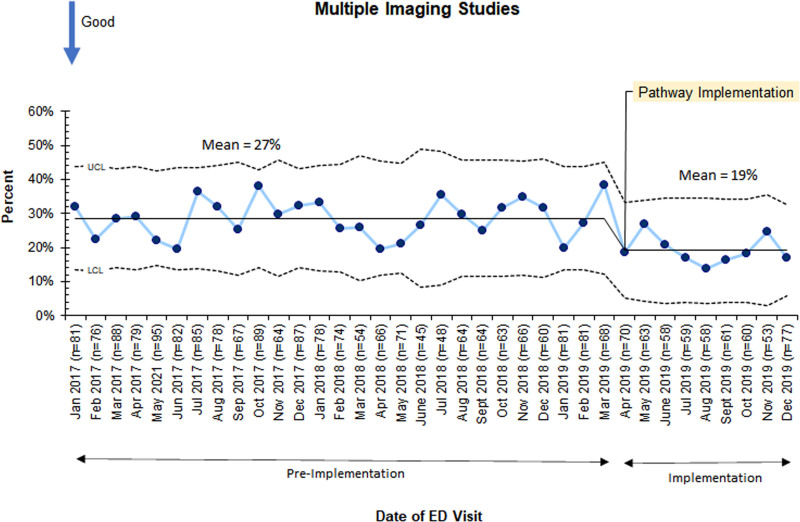
P-chart depicting the trend in the monthly average proportion of patients receiving multiple imaging. LCL, lower control limit; UCL, upper control limit.

When we evaluated the impact of the imaging pathway on all patients receiving a diagnostic evaluation for appendicitis, including all PAS groups, there was a reduction in patients with multiple imaging studies from 29% to 19%. This observed change met special cause variation criteria with eight consecutive points below the centerline following implementation in April 2019 (Fig. 5). Computed tomography (CT) utilization was unchanged (5.7% preimplementation versus 4.4% postimplementation, *P* = 0.85).

The MRI rate increased significantly from 26.2% to 46.9% (*P* < 0.0001) after pathway implementation primarily because the number of MRI studies performed in the intermediate-PAS group increased significantly. ED throughput was not affected by the increased number of MRIs, as room to disposition time did not increase after pathway implementation (**see Figure 2, Supplemental Digital Content 4**, which describes ED average room to disposition time. X-bar chart showing average room to disposition time (minutes) in the ED for workup in suspected appendicitis, http://links.lww.com/PQ9/A366).

The negative appendectomy rate pre- post-pathway was unchanged (5.2% versus 2017 historical rate of 4.6%; *P* = 0.83). There were three cases of missed appendicitis postimplementation (1.0%), which was similar to 2017 (1.2%, *P* = 0.82).

Overall imaging costs pre and postimplementation for CT/US/MRI increased from $24,255 to $31,082 per 100 patients.

## DISCUSSION

Imaging stewardship in children with suspected appendicitis has been the focus of multiple QI interventions, but this report is the first to describe a hybrid MRI/US imaging protocol. Our study highlights that the performance of US varies widely, and, even at a dedicated pediatric institution, the diagnostic rate can be very low, particularly in patients with minimal clinical suspicion for appendicitis. We have also shown that imaging stewardship is key, as imaging is not warranted in many low-risk patients. Still, it is challenging to change the culture in the ED and increase ED provider comfort with foregoing imaging in a subset of patients with abdominal pain. This report confirms that a limited, nonsedated appendix MRI is a quick, accurate, and radiation-free alternative with excellent diagnostic accuracy across all PAS groups for those who require imaging.

The diagnostic accuracy of US varies widely with patient factors such as obesity and retrocecal location of the appendix negatively affecting the accuracy.^16,17^ Institutional factors such as the relative experience of US technicians is associated with lower diagnostic accuracy.^6,7,18^ US reporting templates can improve diagnostic accuracy. We adopted such a template at our institution about 4 years before initiating the imaging protocol described in this study. We did not see any improvement in diagnostic US accuracy. It is clear that the US has significant limitations in diagnosing appendicitis and that relying solely on the US may result in missed appendicitis or negative appendectomy. Our pathway would be optimally used by institutions that have been unable to improve their US performance and have ready access to MRI.

Our algorithm encourages the use of up-front MRI in patients who are likely to have a nondiagnostic US. An alternative diagnosis was discovered in 18% of our patients, similar to the reported literature.^9,19^ This is of particular importance in female patients, who have a broader differential diagnosis for RLQ pain, including ovarian pathology. MRI diagnostic accuracy for appendicitis is similar to or better than CT.^9–11^ Disadvantages of MRI include cost, availability, study duration, and the need for sedation in patients unable to lie still. Improvements aimed at these problems include a faster appendix-specific MRI that can be completed in 10–15 minutes.^10^ At our hospital, we have successfully completed MRI studies without sedation in patients as young as 2 years.

The importance of avoiding imaging altogether in a subset of patients with abdominal pain should be emphasized. Less than 1% of patients with a low PAS had appendicitis in our retrospective review. Thus, we determined that this cohort of patients did not warrant any imaging for appendicitis, consistent with other published data.^20,21^ US performed best in the high-PAS group, so these patients had US as their initial imaging study. The intermediate-PAS group generally accounts for the most patients (55.6% in our study), so imaging stewardship efforts must focus on this group. Likely, many patients in the intermediate-PAS group do not require imaging, as 66% of these patients were discharged from the ED, and only 18% had appendicitis. Even though the pathway encouraged discharge of low-risk intermediate PAS patients without any imaging, only 18% of this group did not receive imaging. Despite significantly reducing the number of studies performed in the low-PAS group, our goal to eliminate imaging in this group was not achieved.

A recently published randomized trial to reduce imaging in children with abdominal pain across 17 EDs encountered similar provider reluctance to discharge patients home without imaging.^22^ To address this concern, it is vital to remove the stigma associated with a patient returning to the ED with appendicitis who was previously seen and discharged home with return precautions. This is an acceptable outcome, as the diagnosis can be difficult to make when patients present soon after symptoms begin. A trial of observation (at home or in the hospital) is a very reasonable choice. Collectively, our multidisciplinary group continues to increase awareness of these acceptable outcomes while attempting to minimize unnecessary imaging and encouraging observation when applicable.

It is important to consider cost when assessing the impact of any clinical pathway, and we observed a slight increase in overall imaging costs in the postimplementation period. Jennings et al.^23^ published a cost-effectiveness analysis of a hypothetical group of patients and reported that the optimal strategy for moderate-risk patients (15%–95% probability of appendicitis) was initial US with CT, if the appendix was not visualized but secondary signs of inflammation were present. Although we observed increased costs immediately following implementation, we believe potential cost savings with our pathway exist. These include eliminating all imaging in low-PAS patients, eliminating US in intermediate-PAS patients who are likely to have a nondiagnostic study, and reducing imaging in the group of patients with an intermediate PAS whose symptoms improve while in the ED. We predict these savings can be realized as ED physicians become more comfortable avoiding imaging in the lower-risk groups (low PAS and intermediate PAS whose abdominal pain improves after fluid bolus); however, this culture change will take time. Although we did not fully achieve our SMART aims, we did trend toward our targets.

Our study has several limitations. We report a single-center experience in a freestanding children’s hospital, and generalizability may be limited for several reasons. Our hospital has access to both 24-hour MRI, pediatric radiologists, and pediatric-trained ultrasonography technicians, which are not available at many institutions. Our negative appendectomy rate was about 5% both pre and postimplementation. This high negative appendectomy rate appears anomalous at our institution, as our NSQIP semiannual reports between 2017 and 2020 show that we are a low outlier at 2%, and this imaging protocol has remained in place continuously throughout 2020. The project was not undertaken to reduce negative appendectomy rate, but we wanted to ensure there was not an unintentional increase in the negative appendectomy rate as a balancing measure. Finally, our US performance is below what others report.^16,18^ Increasing the diagnostic accuracy of US would reduce the need for axial imaging after US, and this approach is more cost-effective than going directly to MRI. The protocol described here would be most beneficial for similar organizations that have struggled to improve US performance but have ready access to MRI. Data gathered throughout this QI project have led to initiatives to strengthen our US capabilities.

## CONCLUSIONS

Many centers face significant imaging stewardship obstacles for patients with suspected appendicitis, and we propose a potential solution. The cost of MRI and increasing ED provider comfort levels in forgoing imaging altogether in certain patients are significant determinants of sustainability. Further studies with ongoing cost analyses are necessary to determine the long-term impact of this pathway on resource utilization.

## ACKNOWLEDGMENTS

We acknowledge James Gearhart, Rajiv Rangarajan, and Amy Hemphill (Nemours Children’s Health, Wilmington, DE 19803) for their help with project management, data collection, data analysis, statistical calculations, and dashboard design. We thank Theresa Michel for her assistance with manuscript preparation.

## DISCLOSURE

The authors have no financial interest to declare in relation to the content of this article.

## Supplementary Material


