# Supplementary material for: A Standardized Diagnostic Pathway for Suspected Appendicitis in Children Reduces Unnecessary Imaging
Source: Pediatr Qual Saf. 2022 Mar 30;7(2):e541. doi: 10.1097/pq9.0000000000000541 (PMC8970092; doi:10.1097/pq9.0000000000000541)
Supplement: Supplementary file 1 [file pqs-7-e541-s001.pdf]

A Standardized Diagnostic Pathway for Suspected Appendicitis in Children Reduces Unnecessary Imaging; D'Cruz RJ et al.

Supplemental Digital Content Table 1: Clinical outcomes (2017) for patients stratified by Pediatric Appendicitis Score.

**Pediatric Appendicitis Score (PAS)**	**Patients undergoing imaging (%)**	**Discharged from ED**	**OR for appendectomy**	**Admit for Observation**	**Nonoperative management**	**Admit with alternate diagnosis**	**OR other indication**	**Negative appendectomy**	**Pathology confirmed appendicitis**
**Low (1-3)**	268 (28%)	214 (79.9%)	3 (1.1%)	7 (2.6%)	0 (0%)	38 (14.2%)	6 (2.2%)	1 (33.3%)	2 (66.7%)
**Intermediate (4-6)**	542 (56%)	340 (56%)	83 (15.3%)	18 (3%)	4 (0.7%)	82 (15.1%)	15 (2.8%)	6 (7.2%)	77 (92.8%)
**High (7-10)**	161 (17%)	42 (26%)	87 (60%)	5 (3%)	10 (6.2%)	17 (10.6%)	0 (0%)	1 (1.1%)	86 (98.9%)

ED, emergency department; OR, operating room
