# Supplementary material for: A Standardized Diagnostic Pathway for Suspected Appendicitis in Children Reduces Unnecessary Imaging
Source: Pediatr Qual Saf. 2022 Mar 30;7(2):e541. doi: 10.1097/pq9.0000000000000541 (PMC8970092; doi:10.1097/pq9.0000000000000541)
Supplement: Supplementary file 2 [file pqs-7-e541-s002.pdf]

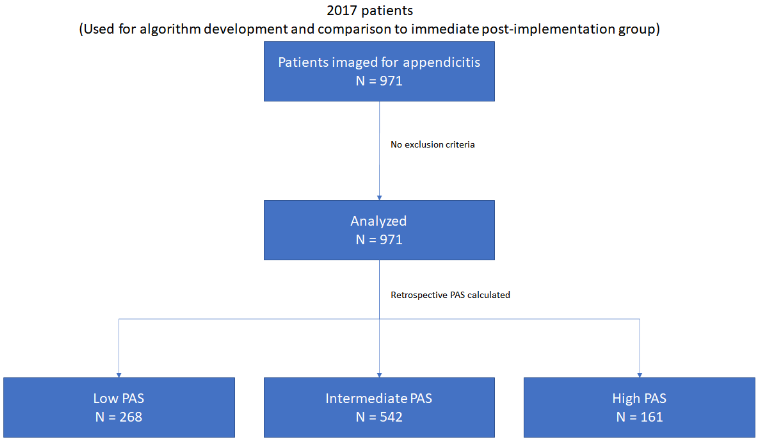
2017 patients
(Used for algorithm development and comparison to immediate post-implementation group)
Patients imaged for appendicitis
N = 971
No exclusion criteria
Analyzed
N = 971
Retrospective PAS calculated
Low PAS
N = 268
Intermediate PAS
N = 542
High PAS
N = 161

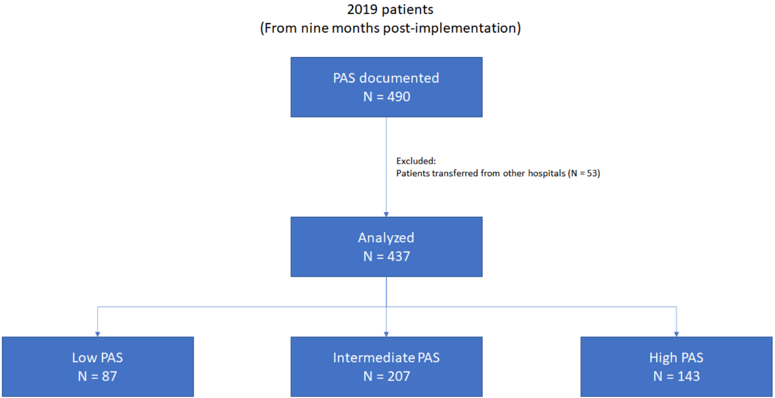
2019 patients
(From nine months post-implementation)
PAS documented
N = 490
Excluded:
Patients transferred from other hospitals (N = 53)
Analyzed
N = 437
Low PAS
N = 87
Intermediate PAS
N = 207
High PAS
N = 143
