# Supplementary material for: A Standardized Diagnostic Pathway for Suspected Appendicitis in Children Reduces Unnecessary Imaging
Source: Pediatr Qual Saf. 2022 Mar 30;7(2):e541. doi: 10.1097/pq9.0000000000000541 (PMC8970092; doi:10.1097/pq9.0000000000000541)
Supplement: Supplementary file 3 [file pqs-7-e541-s003.pdf]

A Standardized Diagnostic Pathway for Suspected Appendicitis in Children Reduces Unnecessary Imaging; D’Cruz RJ et al.

Supplemental Digital Content Table 2: Comparison of patient characteristics in the pre-implementation (2017) and post-implementation (April – December 2019) groups.

Feature	Pre-implementation (2017)	Post-implementation (April – Dec 2019)	p value
**Age**			0.39
Mean (IQR)	10.9 (7.8 - 14.4)	11.1 (8.2 - 14.3)	
**Weight**			0.47
Mean (IQR)	43.4 (26.9 - 57.4)	44.2 (27.6 - 57)	
**Sex (%)**			0.37
Male	444 (45.7%)	211 (48.3%)	
Female	527 (54.3%)	226 (51.7%)	
**Race (%)**			0.69
White	644 (66.3%)	278 (63.6%)	
Black/African American	131 (13.5%)	68 (15.6%)	
Other	193 (19.9%)	89 (20.4%)	
Refused	3 (0.3%)	2 (0.5%)	

IQR, interquartile range
