# Supplementary figures and images for: A Standardized Diagnostic Pathway for Suspected Appendicitis in Children Reduces Unnecessary Imaging
Source: Pediatr Qual Saf. 2022 Mar 30;7(2):e541. doi: 10.1097/pq9.0000000000000541 (PMC8970092; doi:10.1097/pq9.0000000000000541)

## ED Visit Duration

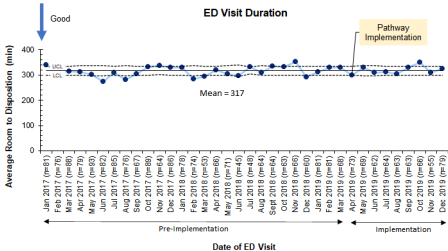

Supplement: Supplementary file 4 [file pqs-7-e541-s004.pdf]
